# In-Needle Pre-Column Derivatization for Amino Acid Quantification (iPDAQ) Using HPLC

**DOI:** 10.3390/metabo12090807

**Published:** 2022-08-28

**Authors:** Yuki Soma, Yoshihiro Izumi, Takehiko Shimohira, Masatomo Takahashi, Yuri Imado, Saki Tominaga, Kanako Tokito, Kosuke Hata, Shoji Shinadama, Mana Oshiro, Yoshihiro Hayakawa, Takeshi Bamba

**Affiliations:** 1 Division of Metabolomics/Mass Spectrometry Center, Medical Research Center for High Depth Omics, Medical Institute of Bioregulation, Kyushu University, 3-1-1 Maidashi, Higashi-ku, Fukuoka 812-8582, Japan; 2 Laboratory for Synthetic Biology, Graduate School of Bioresource and Bioenvironmental Sciences, Kyushu University, W5-729, 744 Motooka, Nishi-ku, Fukuoka 819-0395, Japan; 3 Shimadzu Corporation, 1, Nishinokyo-Kuwabara-cho, Nakagyo-ku, Kyoto 604-8511, Japan

**Keywords:** HPLC, amino acids quantification, pre-column derivatization, SRM1950

## Abstract

Pre-column fluorescent derivatization has been used for the fast quantification of amino acids using high-performance liquid chromatography (HPLC) systems. However, it generally requires an offline in-vial derivatization process with multiple derivatization reagents. The offline derivatization requires the same number of reaction vials as the number of sample vials for use as a reaction chamber for the derivatization reaction in an autosampler. Therefore, the number of samples analyzed per batch using the pre-column derivatization method is halved. To benefit from the pre-column derivatization method, we transformed the derivatization process from an offline chamber process to an online in-needle process (in-needle Pre-column Derivatization for Amino acids Quantification; iPDAQ). Fluorescent derivatization in the injection needle obviated the need for vacant vials as reaction chambers. Consequently, the throughput per batch improved up to two times, and the consumption of derivatization reagents was reduced to less than one-tenth of that in the conventional vial method. We demonstrated to separate and quantify the amino acids in various biological samples. Herein, we presented a novel HPLC-based amino acid quantification method that enables the continuous analysis of a large number of samples. The iPDAQ facilitates accurate amino acid quantification due to the automation of derivatization and achieves improvement in the throughput and reduction of analysis labor.

## 1. Introduction

Amino acids are important nutrients for every organism, because they are the building blocks of proteins, metabolic intermediates, and substrates for energy production. Abnormalities of amino acids are observed in a broad spectrum of inherited metabolic diseases (e.g., disorders of amino acid metabolism and transport, organic acidemias, and ureagenesis defects) [1]. Amino acid deficiency has long been known to impair immune function and increase susceptibility to infectious diseases in animals and humans [2]. For clinical application, a network analysis of amino acid concentration in plasma and tissue to generate an amino index has been developed for potential diagnostic use [3]. There is a huge demand for amino acid quantification for medical and clinical applications. In addition to their importance as nutrients, amino acids are components that affect the taste of foods. Therefore, there is also a great demand for the quantification of amino acids in the food industry. Since the availability of amino acids in the environment significantly affects the growth and the metabolism of microbes, the amino acid concentration can be valuable information for foods and chemical industries utilizing microbial fermentation. As highlighted above, there is a definite demand for the development of methods to quantify amino acids.

Cation-exchange chromatography coupled with online post-column derivatization is currently one of the most common analytical methods for amino acid quantitation. Examples of combinations of post-column derivatization and the detection methods are ninhydrin derivatization followed by spectrophotometric detection [4] and *o*-phthalaldehyde (OPA) derivatization followed by fluorescence detection [5]. Although these methods facilitate high-chromatographic separation performance and quantitative accuracy for amino acids, they require a long analysis time of 1 h or more for each sample analysis.

On the contrary, reverse-phase (RP) high-performance liquid chromatography (HPLC) with fluorescence detection using pre-column derivatization has been developed as a rapid quantitative analysis for amino acids [6,7,8]. The most studied fluorescent derivatization reagent for amino acids is OPA, but it cannot react with secondary amino acids, such as proline [8]. For the analysis of secondary amino acids, 9-fluorenylmethyl chloroformate (FMOC) has been studied. The OPA/FMOC derivatization allows the simultaneous quantification of primary and secondary amino acids [9]. Furthermore, the derivatization of amino acids using OPA and 3-mercaptopropionic acid (MPA) has been developed, which results in the formation of more stable and hydrophobic amino acid derivatives than OPA derivatization [10,11,12], enhancing the quantitative performance and separation of derivatives with RP-HPLC. For these reasons, the methods for the simultaneous quantification of primary and secondary amino acids have been developed using OPA/MPA/FMOC fluorescent derivatization for the pre-column derivatization of amino acids [13,14,15] (Figure S1).

**Figure 1:**
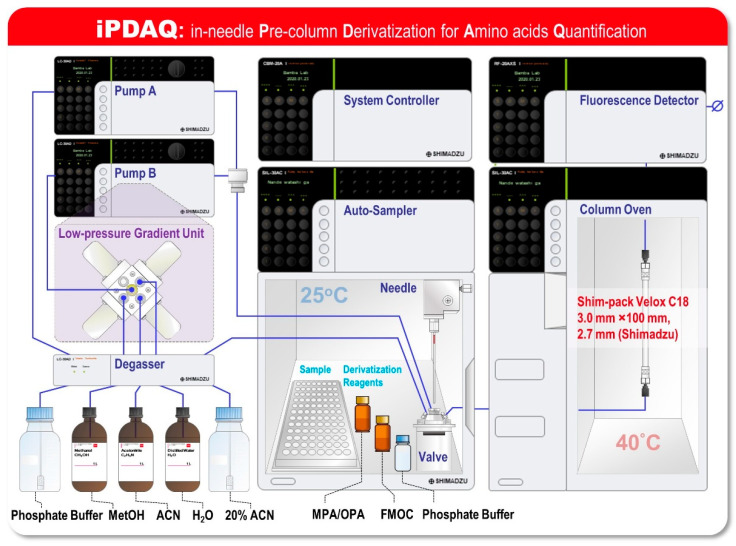
Schematic of the system configuration of an in-needle Pre-column Derivatization system for Amino acid Quantification (iPDAQ). The diagram shows an HPLC system for amino acid quantification based on the method of the fast pre-column derivatization of amino acids, which facilitates the automated pre-column derivatization of amino acids using an autosampler.

Although the pre-column derivatization-based RP-HPLC methods facilitate a faster analysis than that achieved with cation-exchange chromatography coupled with online post-column derivatization methods, they require offline derivatization with multiple derivatization reagents. To ensure the quantitative performance of this method, automation of the “just-in-time” derivatization reaction overlapping the HPLC analysis is required to align the reaction time of derivatization for all samples. In the pre-column derivatization, an overlapping HPLC analysis has been employed, thus far, for practical use—the same number of reaction and sample vials is prepared as a reaction chamber for derivatization in the autosampler [13,14,15]. Since the number of samples that can be set in the autosampler is halved for the abovementioned reason, the advantage of a pre-column derivatization system as a high-speed analysis has been limited. We surmised that transformation of the derivatization process from an offline chamber process to an online process would be helpful to leverage the advantages of the pre-column derivatization of amino acids using OPA/MPA/FMOC. To confirm this, we designed an in-needle Pre-column Derivatization for Amino acid Quantification (iPDAQ) as an online OPA/MPA/FMOC derivatization method (Figure 1). For fluorescent derivatization in the injection needle of an autosampler, vacant vials as reaction chambers are not required. Additionally, the amount of derivatization reagents required for the microliter scale injection needle is expected to be reduced compared to that in the conventional vial method. We investigated several system parameters of iPDAQ and performed a quantitative analysis of the amino acids in the biological samples. In this study, we presented a novel HPLC-based analytical method that enables the accurate, high-throughput and labor-less quantification of amino acids using online in-needle fluorescent derivatization for the first time.

## 2. Results and Discussion

### 2.1. Development of the iPDAQ Method

**Figure 2:**
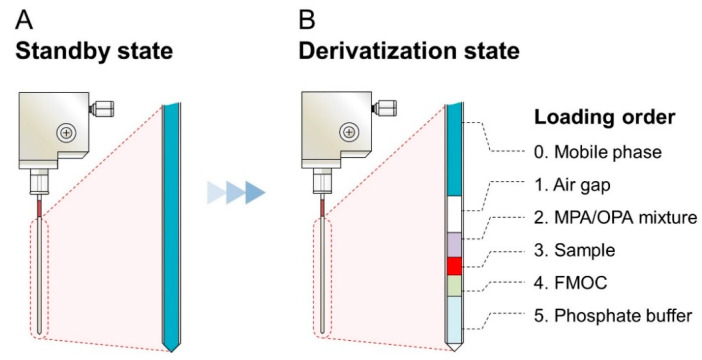
Diagram showing the contents of the needle with the amino acid derivatization using the iPDAQ. (**A**) State of the needle before the start of the derivatization procedure. (**B**) Function of the air gap and the loading order of the sample and derivatization regents.

For the development of the iPDAQ, we employed OPA/MPA derivatization for the primary amino group (Figure S1A) and FMOC derivatization for the secondary amino group (Figure S1B). A two-step derivatization was performed in the injection needle of the autosampler. Since the needle of the autosampler was filled with mobile phase at the initial stage (Figure 2A), an air gap between the mobile phase and sample/derivatization reagents was made as the first step of the procedure to prevent the contamination of the mobile phase with the samples (Figure 2B). The condition of the automated sample derivatization procedure was determined as shown in Table S1. Details of the reagents and analyte concentration are given in Table S2. With this method, all derivative reaction solutions loaded into the needle were injected for HPLC analysis by direct injection.

**Figure 3:**
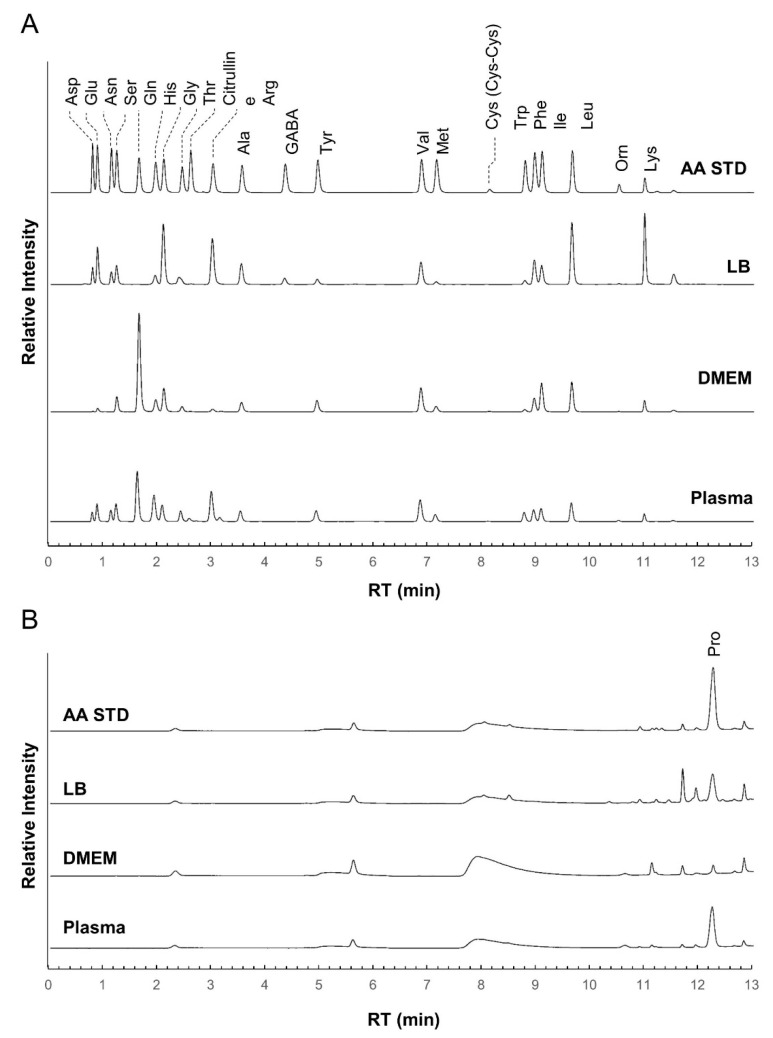
Chromatogram obtained using the iPDAQ method. Each panel shows chromatograms of the amino acid standard mixture (STD), Luria–Bertani medium (LB), Dulbecco’s modified Eagle’s medium (DMEM), and human plasma extract (Plasma). (**A**) The chromatogram for the primary (λEx = 350 nm/λEm = 450 nm) (**B**) The chromatogram for secondary (λEx = 266 nm/λEm = 305 nm) amino group. The horizontal axis is the retention time (RT), and the vertical axis is the relative intensity.

At first, we checked the separation performance of the iPDAQ using a mixture of standard substances (STD) of 23 amino acids (alanine (Ala), arginine (Arg), asparagine (Asn), aspartic acid (Asp), citrulline, cystine (Cys-Cys), γ-aminobutyric acid (GABA), glutamine (Gln), glutamic acid (Glu), glycine (Gly), histidine (His), isoleucine (Ile), leucine (Leu), lysine (Lys), methionine (Met), ornithine (Orn), phenylalanine (Phe), proline (Pro), serine (Ser), threonine (Thr), tryptophan (Trp), tyrosine (Tyr), and valine (Val). All target amino acids were clearly separated and detected with the iPDAQ using a 10 μM STD mixture (Figure 3). The repeatability of the iPDAQ method was investigated by analyzing the 10 μM STD mixture 24 times (Figure S2). All target amino acids were separated with small fluctuations in the retention time (relative standard deviations (RSDs) = 0.1–0.7%). The iPDAQ also showed sufficient repeatability of the detection intensity (peak area) as a HPLC quantitative method (RSDs = 1.3–6.5%). Next, we applied the iPDAQ to several biological samples (Luria–Bertani (LB) medium, Dulbecco’s modified Eagle’s medium (DMEM), and human plasma). All target amino acids were well-separated regardless of the background matrix in each sample (Figure 3).

Using the iPDAQ, the consumption amount of each derivatization reagent was reduced compared with that in the conventional vial method. An example of the conventional vial method is shown in Table S3. Compared with this case, the consumption of MPA/OPA and FMOC was reduced by 97 % and 90%, respectively, in the iPDAQ method. Moreover, the upper limit of the sample size for one analytical batch with the iPDAQ (192 samples per batch) was improved 4.3-fold compared with that in the conventional vial method (45 samples per batch). These results clearly demonstrate that iPDAQ facilitates amino acid quantification and reduces the analytical cost and labor.

### 2.2. System Validation of the Optimized iPDAQ

Next, we obtained the parameters for the separation and quantification performance of iPDAQ with the standard substances of 23 amino acids to further evaluate the iPDAQ method (Table 1). For all amino acids, the linearity of the calibration curves was ensured over the range from 0.05 to 50 μM (Table 1). The limit of detection (LOD) for each amino acid is listed in Table 1. Such detection sensitivity and quantification performance of the iPDAQ method was comparable to the general GC/MS-based amino acids analysis using single-quadrupole GC/MS [16,17,18]. Moreover, we performed the addition–recovery test for all amino acids for validation of the quantification performance of iPDAQ. As shown in Table 1, the recovery ratios were good for all amino acids (88–105%). These results clearly demonstrate that iPDAQ is a high-quality amino acid quantification system. Nevertheless, there might be room to improve the detection sensitivity and quantification performance through the optimization of the derivatization condition (e.g., derivatization reagents concentration, reaction time, reaction temperature, and in-needle mixing of the derivatization solution).

**Figure 4:**
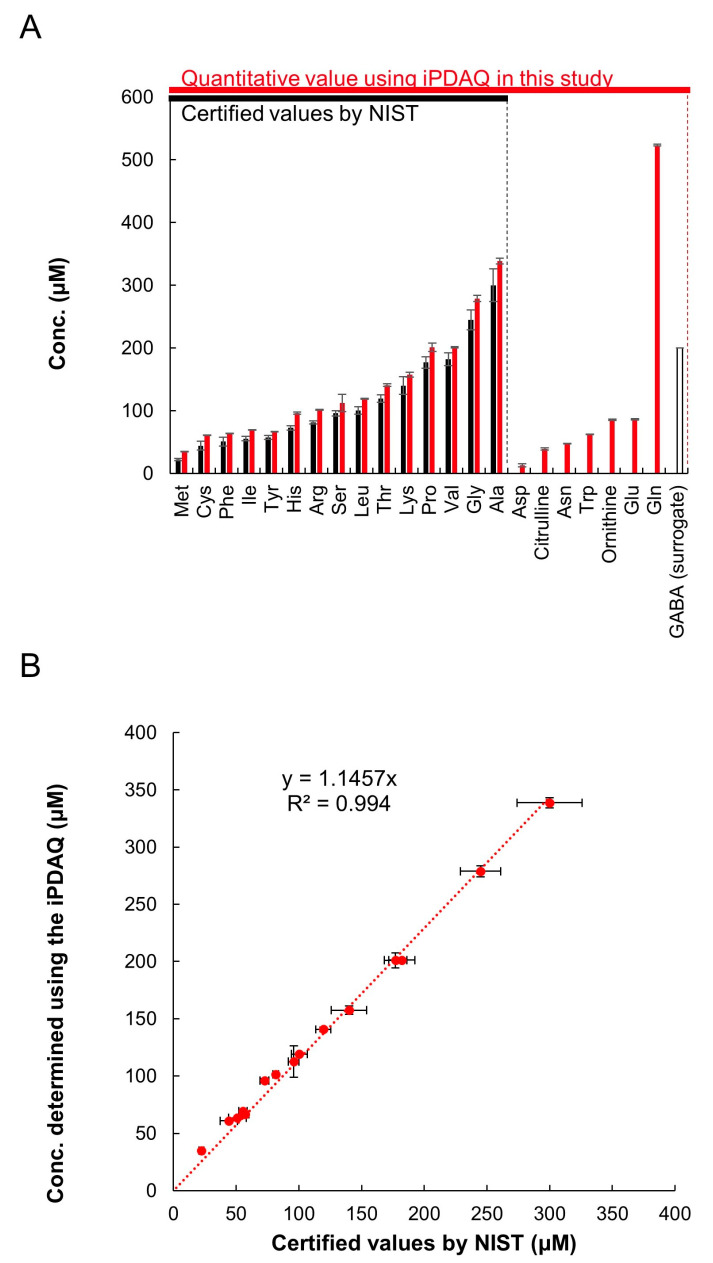
iPDAQ method validation using reference human plasma sample SRM1950. (**A**) Amino acid concentration in SRM1950. Black bars, certified amino acid concentrations provided by NIST. Red bars, amino acid concertation quantified using the iPDAQ method. Open bar, GABA used as a surrogate. (**B**) Comparison of the amino acid concentrations determined using the iPDAQ with the certified values provided by NIST. Error bars indicate the standard deviation (*n* = 3).

For further validation of the method, we quantified the amino acids in a reference human plasma sample (SRM1950) provided by the National Institute of Standards and Technology (NIST, Gaithersburg, MD, USA). SRM1950 was provided with several metabolites quantified using various methods appropriate for each target metabolite (referred to as certified values; https://www-s.nist.gov/srmors/view_detail.cfm?srm=1950, accessed on 22 July 2022). NIST provides the certified values of 15 amino acids in SRM1950 (Met, Cys, Phe, Ile, Tyr, His, Arg, Ser, Leu, Thr, Lys, Pro, Val, Gly, and Ala), which are shown as blue bars in Figure 4A. We compared the certified values and quantification results employing the iPDAQ method. For plasma extraction and amino acid quantification, we used GABA as a surrogate standard, because it was not detected in SRM1950 (Figure 3A). Using the iPDAQ method, we succeeded in quantifying 22 amino acids in the SRM1950 extract with high repeatability (red bars in Figure 4A). As shown in Figure 4B, the concentrations determined by us and the certified values provided by NIST for the 15 amino acids were almost equivalent. These results prove the accuracy and repeatability of amino acid quantification using the iPDAQ method.

### 2.3. Time Course Monitoring of Amino Acids in Cell Culture Medium

We demonstrate an application of the iPDAQ method in biological research. We performed a time course analysis of the amino acid concentration in samples of the culture medium used for culturing HeLa cells. For this experiment, we prepared two types of samples—one was a typical HeLa cell culture in Dulbecco’s modified Eagle’s medium (DMEM; control), and the other was HeLa cells cultured in DMEM containing an inhibitor of amino acid transporter BCH (2-aminobicyclo-(2,2,1)-heptane-2-carboxylic acid) (BCH addition). BCH is a selective and competitive inhibitor of L-type amino acid transporter 1.

**Figure 5:**
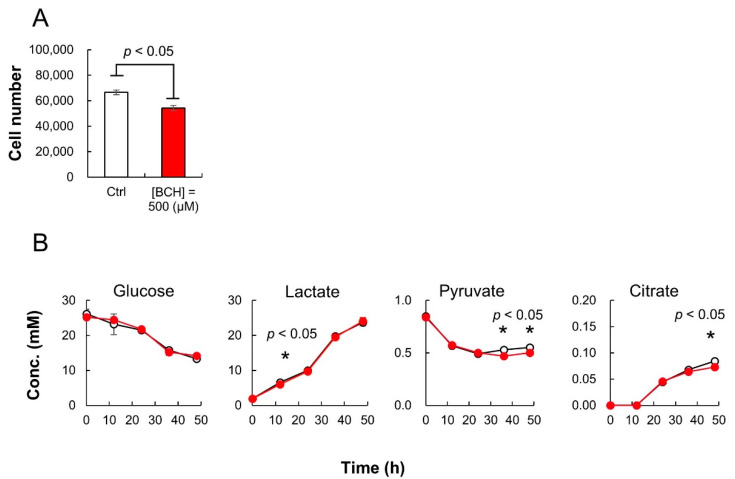
HeLa cell culture profiles with or without 2-aminobicyclo-(2,2,1)-heptane-2-carboxylic acid (BCH). (**A**) Cell number after 48-h cultivation with (red; (BCH) = 500 μM) or without (white; control) BCH addition. (**B**) Time course of sugar and organic acid concentration in culture medium. White and red circles indicate the results for cultures without and with BCH addition ((BCH) = 500 μM), respectively. Error bars indicate standard deviation (*n* = 3).

During the 48-h cultivation of HeLa cells, 500 μL of culture medium was collected every 12 h for both the sample groups. The collected medium was diluted 100 times with sterilized distilled water and subsequently analyzed using iPDAQ and other HPLC analysis systems for organic acids and sugars. The growth of HeLa cells was inhibited by BCH (Figure 5A). Although BCH inhibited citrate production, there was no significant difference in glucose consumption and lactate production (Figure 5B). The pyruvate consumption by HeLa cells upon BCH addition was rather enhanced compared with that in the cells cultured in the absence of BCH (Figure 5B).

**Figure 6:**
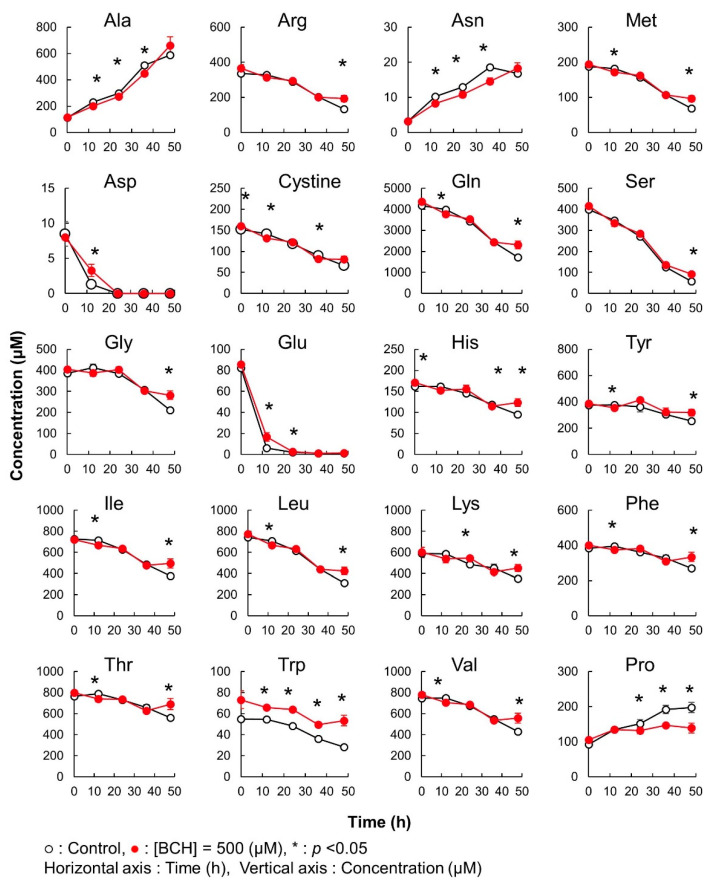
Method validation of iPDAQ: time course of the amino acid concentration in the medium used for the cultivation of HeLa cells. Each graph represents the time course of the amino acid concentration. Close and open circles indicate the results with (BCH addition) and without (Control) the amino acid inhibitor. Error bars indicate the standard deviation (*n* = 3).

In this case, significant differences in the amino acid concentration were observed between cultures without and with BCH addition (Figure 6). In both the cases, all amino acids, except Ala, Asn, and Pro, were consumed with the progress of culture. Ala, Asn, and Pro were exported to the culture medium in both the conditions. Among the consumed amino acids, Gln and Asp were completely depleted by 36 h. However, the consumption of Gln and Asp was significantly attenuated by the addition of BCH. Additionally, the amounts of Ala, Asn, and Pro exported in samples with a BCH addition were significantly lower than those in samples not treated with BCH from 12 to 36 h of culture. These results clearly demonstrate that BCH inhibited the transportation of some amino acids.

These results indicate that BCH affects the amino acid metabolism but not glucose consumption, followed by glycolysis. On the contrary, it caused the excess consumption of extracellular pyruvate, which might compensate for amino acid consumption. The inhibition of cell growth upon BCH addition was clearly caused by the inhibition of amino acid transport. Here, we prove the utility of the iPDAQ and other HPLC quantification systems for monitoring the detailed profiles of changes in the composition of the medium used for cell cultivation. In particular, the iPDAQ method, which is highly sensitive for the quantitative analysis of amino acids, revealed significant differences within the ranges for trace amino acid concentrations.

## 3. Materials and Methods

### 3.1. Chemicals and Reagents

All reagents used in this study were purchased from Nacalai Tesque Inc. (Kyoto, Japan) unless otherwise noted. Amino acid standard substances were prepared based on an Amino Acids Mixture Standard Solution Type H (High Range), including L-Asp, L- Glu, L-Ser, L-His, Gly, L-Thr, L-Arg, L-Ala, L-Tyr, L-Val, L-Met, L-Cys-Cys, L-Phe, L-Ile, L-Leu, L-Lys, and L-Pro (2.5 mM each), which was provided by FUJIFILM Wako Pure Chemical Corp. (Osaka, Japan). The amino acid standard substances mixture (AA std mix) was prepared by the addition of individual stock solutions of L-Asn, L-Gln, L-Trp, GABA, L-Orn, and L-citrulline (2.5 mM each).

Luria–Bertani medium (Miller) (LB medium) was purchased from Nacalai Tesque Inc. DMEM was purchased from Thermo Fisher Scientific, Inc., (Waltham, MA, USA). FBS and 1% *v/v* penicillin–streptomycin solution were purchased from Thermo Fisher Scientific, Inc. NIST SRM1950 plasma was supplied by Sigma-Aldrich (St. Louis, MO, USA).

### 3.2. Preparation of LB Medium, DMEM, and Human Plasma Extract

LB medium was prepared by dissolving 25 g of LB medium powder (Nacalai Tesque Inc.) in 1 L of water and autoclaving. The medium was 100-fold diluted with water for the iPDAQ analysis. DMEM supplemented with 10% *v/v* FBS (Thermo Fisher Scientific, Inc., Waltham, MA, USA) and 1% *v/v* penicillin–streptomycin solution (Thermo Fisher Scientific, Inc.) was 100-fold diluted with water for the iPDAQ analysis. Extraction of human plasma (SRM 1950) was performed using the Bligh and Dyer method [19], with some modifications as follows: 50 µL of plasma and 950 µL of cold methanol were mixed in a 2-mL microtube by vortexing for 1 min; the mixture was sonicated for 5 min and then centrifuged at 16,000× *g* for 5 min at 4 °C. The supernatant (400 μL) was collected in a clean microtube, and 380 μL of chloroform and 284 μL of water were added to it. After gentle vortexing, the aqueous and organic phases were separated by centrifugation at 16,000× *g* for 5 min at 4 °C. The aqueous (upper) layer (300 μL) was transferred to a clean tube and evaporated under vacuum. The dried extract was reconstituted in 50 μL of water/acetonitrile (1:1, *v/v*) and stored at –80 °C until the analysis. The human plasma extract was 10-fold diluted with water for the iPDAQ analysis.

### 3.3. Derivatization Reagents

Derivatization reagents for the iPDAQ were prepared as follows: 0.1 M borate buffer was prepared by dissolving 0.62 g borate and 0.20 g NaOH in 100 mL water. MPA stock solution (0.1% MPA) was prepared in 0.1 M borate buffer. OPA (10 mg) was dissolved in 0.3 mL methanol by sonication, and then, 0.7 mL of 0.1 M borate buffer and 4 mL of water were added, resulting in a 2 mg/L OPA stock solution in a mixture of water/methanol/borate (3/7/40). The FMOC stock solution was prepared by dissolving 10 mg of FMOC in 25 mL acetonitrile. Acidic phosphate solution (0.1 M) was prepared by dissolving 0.68 g KH_2_PO_4_ and 85% phosphoric acid in 100 mL water. After the preparation, the reagents were dispensed in 1 mL clean vials and stored at –30 °C until use.

### 3.4. In-Needle Derivatization of Amino Acids

Amino acid quantification by the iPDAQ was performed using HPLC comprising LC-30AD, SIL-30AC, CTO-20AC, and RF-20Axs (Shimadzu Co., Kyoto, Japan) equipped with a reverse phase column (Shim-pack Velox C18, 3.0 mm × 100 mm, 2.7 μm, Shimadzu Co.) based on automated pre-column derivatization with OPA/MPA and FMOC. Derivatization and injection conditions in the iPDAQ are summarized in Tables S1 and S2. The pre-column derivatization using OPA/MPA and FMOC was performed automatically using an autosampler (Shimadzu SIL-30AC). The MPA/OPA mixture was pre-mixed in a clean vial using 1 mL of 0.1% MPA stock solution and 0.5 mL of 2 mg/L OPA stock solution. As the first step, 1.5 μL of the MPA/OPA mixture was loaded using an injection needle after introducing a 2.5-μL air gap between the reagent and rinse solution. After rinsing the outside of the needle with 20% acetonitrile, 0.5 μL of the samples were loaded, and the loaded solution was mixed by needle pumping (10 times). After an incubation of 0.5 min, 0.5 μL of 0.4 g/L FMOC stock solution was loaded, followed by repeating suction and discharge in the needle (10 times pumping) for mixing. After further incubation for 2 min, the same volume of 0.1 M acidic phosphate buffer was loaded with the total reactant (2.5 μL). A total 2.5 μL of the solution was injected into the HPLC system by direct injection. Samples were separated using a binary nonlinear gradient with mobile phase A (2.04 g KH_2_PO_4_, 0.87 g K_2_HPO_4_ per 1 L water) and mobile phase B (water:acetonitrile:methanol = 15:45:40 by volume). The elution conditions were as follows: equilibration (4 min, 9% B); gradient (9% from 0 to 0.5 min, 9–12% from 0.5 to 1 min, 12% from 1 to 1.5 min, 12–22% from 1.5 to 6 min, 22–30% from 6 to 8 min, 30–53% from 8 to 10.5 min, 53% from 10.5 to 12.5 min, and 53–100% from 12.5 to 13 min); and cleaning (3 min, 100% B). The flow rate was adjusted to 1 mL/min, and the column temperature was maintained at 35 °C. RF-20A was used as a fluorescence detector for derivatized amino acids. The parameter settings for the fluorescence detector were as follows: excitation/emission, 350/450 nm for primary amino acid derivatives and 266/305 nm for secondary amino acid derivatives; cell temperature, 30 °C; and response, 50 ms.

### 3.5. Preparation of Calbration Curve and Quantification of Amino Acids

Calibration curves for 23 amino acids were prepared by analyzing serial dilutions of the AA STD mix (0.05–50 μM) described above. The Amino Acids Mixture Standard Solution Type H (High-Range) was provided by FUJIFILM Wako Pure Chemical Corporation and included 2.5 mM Cys-Cys instead of cysteine (Cys), as Cys gets easily oxidized to form Cys-Cys, a dimer of two cysteine molecules. In the presence of MPA, Cys-Cys is reduced to Cys, followed by fluorescent derivatization [9]. Therefore, the quantitative value was doubled to convert the value obtained from the Cys-Cys calibration curve as the Cys concentration. The AA STD mix (50 μM) was used for the standard addition–recovery test using the DMEM medium of the HeLa cell culture. The DMEM medium for the HeLa cell culture and the AA STD was mixed 1 to 1, and then, this mixture and the original DMEM culture medium were analyzed using the iPDAQ. The recovery ratio was calculated according to Figure S3.

### 3.6. Cultivation of HeLa Cells

HeLa cells (ATCC) were cultured in dishes (diameter, 10 cm) containing 10 mL of DMEM supplemented with 10% FBS and antibiotics. Cultivation dishes were incubated in a water jacket CO_2_ incubator (WCI-165, ASTEC Co., Fukuoka, Japan) under a humidified atmosphere of 5% CO_2_ at 37 °C. HeLa cells were harvested when semiconfluent by treatment with trypsin–EDTA solution and collected in a 15-mL tube. The floating cells in the suspension were counted using a cell counter (Moxi Z, ASONE Co., Osaka, Japan).

### 3.7. Amino Acid Transporter Inhibitor Assay

BCH (500 μM), an inhibitor of the amino acid transporter, was added to the HeLa cell culture. The main culture for the BCH assay of the HeLa cells started with an initial cell number of 1 × 10^5^ cells for both the control and BCH addition conditions. The culture medium (100 μL) was collected every 12 h and centrifuged at 240× *g* for 5 min at 4 °C. The supernatant was collected in a new 1.5-mL microtube and stored at –30 °C until the HPLC analysis of glucose, organic acids, and amino acids.

### 3.8. HPLC Analysis of Glucose and Organic Acids in HeLa Cell Culture Medium

HPLC analyses of glucose and organic acids were performed according to our previous reports [20,21]. Organic acids (α-ketoglutarate, pyruvate, citrate, lactate, acetate, formate, fumarate, malate, and succinate) in the supernatant were quantified using an HPLC system comprising LC-30AD, SIL-30AC, CTO-20AC, and CDD-10AVP (Shimadzu Co.) and equipped with three tandem ion-exclusion chromatography columns (Shim-pack Fast-OA; 7.8 mm × 100 mm; 5 μm; Shimadzu Co.) and a guard column (Shim-pack Fast-OA (G); 4.0 mm × 10 mm; 5 mm; Shimadzu Co.). The samples were separated with a mobile phase consisting of 5.0 mM p-toluenesulfonate. The column temperature was 40 °C, and the flow rate was 0.8 mL/min. CDD-10AVP was used for post-column pH-buffered electrical conductivity detection. Post-column pH was buffered with a pH-buffering solution containing 5.0 mM p-toluenesulfonate, 20 mM Bis-Tris, and 0.1 mM EDTA. For each sample, 5-μL aliquots were injected.

Glucose concentration was determined using an HPLC system comprising LC-30AD, SIL-30AC, CTO-20AC, and RID-10A (Shimadzu Co.) equipped with a ligand exchange chromatography column (ULTRON AF-HILIC-CD; 4.6 mm × 250 mm or 150 mm; 5 μm; Shinwa Chemical Industries Ltd., Kyoto, Japan). The samples were separated with 85% (*v/v*) acetonitrile. The column temperature was 60 °C, and the flow rate was 0.8 mL/min. For each sample, 5-μL aliquots were injected.

## 4. Conclusions

Herein, we present iPDAQ, a novel online in-needle pre-column derivatization system for amino acid quantification using HPLC. Using the iPDAQ, we could achieve a reduction in the amount of derivatization reagents used and an improvement in the throughput, which ensured a high quantification performance. We demonstrated the application of iPDAQ in profiling amino acid changes in the cultivation of cancer cells. This novel method facilitates a reduction in plastic expendables, reagent and sample consumption, and labor incurred by the operator and can contribute to the Sustainable Development Goals (SDGs), especially to Goals 8 (DECENT WORK AND ECONMOMIC GROWTH), 9 (INDUSTRY, INNOVATION AND INFRASTRUCTRE), 12 (RESPONSIBLE CONSUMPTION AND PRODUCTION), and 13 (CLIMATE ACTION). The iPDAQ and its updated systems are expected to contribute to various biological study fields and industries.

## Figures and Tables

**Table 1:** System validation using standard substances.

#	Amino Acid	Abbreviation	KEGG ID	Retention Time (*n* = 3)		LOD *	Linear Range **	*R* ^2^	Recovery Ratio ***
				Mean ± SD	RSD (%)	(μM)	(μM)		(%)
1	L-Aspartic acid	Asp	C00049	0.710 ± 0.003	0.403	0.004	0.05–50	1.000	98.8
2	L-Glutamic acid	Glu	C00025	0.997 ± 0.003	0.296	0.009	0.05–50	0.999	96.7
3	L-Asparagine	Asn	C00152	1.419 ± 0.005	0.372	0.008	0.05–50	0.999	98.8
4	L-Serine	Ser	C00065	1.676 ± 0.005	0.322	0.005	0.05–50	0.999	98.6
5	L-Glutamine	Gln	C00064	2.074 ± 0.005	0.228	0.010	0.05–50	0.999	88.0
6	L-Histidine	His	C00135	2.455 ± 0.007	0.278	0.008	0.05–50	0.999	97.8
7	Glycine	Gly	C00037	2.570 ± 0.007	0.283	0.009	0.05–50	0.999	97.3
8	L-Threonine	Thr	C00188	2.794 ± 0.008	0.275	0.008	0.05–50	0.998	98.9
9	L-Citrulline	Citrulline	C00327	3.108 ± 0.008	0.265	0.006	0.05–50	0.999	100.2
10	L-Arginine	Arg	C00062	3.413 ± 0.008	0.231	0.008	0.05–50	0.999	99.2
11	L-Alanine	Ala	C00041	4.071 ± 0.009	0.219	0.016	0.05–50	0.996	95.5
12	γ-Aminobutyric acid	GABA	C00334	5.199 ± 0.010	0.190	0.009	0.05–50	0.999	97.1
13	L-Tyrosine	Tyr	C00082	5.872 ± 0.011	0.188	0.008	0.05–50	0.999	97.4
14	L-Valine	Val	C00183	8.154 ± 0.008	0.104	0.007	0.05–50	0.999	97.9
15	L-Methionine	Met	C00073	8.291 ± 0.008	0.097	0.008	0.05–50	0.999	93.8
16	L-Cystine	Cys-Cys	C00491	8.842 ± 0.037	0.419	0.124	0.05–50	0.999	98.4
17	L-Tryptophan	Trp	C00078	9.301 ± 0.006	0.068	0.007	0.05–50	0.999	96.9
18	L-Phenylalanine	Phe	C00079	9.501 ± 0.006	0.065	0.006	0.05–50	0.999	105.5
19	L-Isoleucine	Ile	C00407	9.653 ± 0.007	0.069	0.006	0.05–50	0.999	100.4
20	L-Leucine	Leu	C00123	9.945 ± 0.005	0.054	0.006	0.05–50	0.999	100.1
21	L-Ornithine	Orn	C00077	10.185 ± 0.007	0.066	0.043	0.05–50	0.994	92.0
22	L-Lysine	Lys	C00047	10.496 ± 0.008	0.075	0.029	0.05–50	0.996	100.1
23	L-Proline	Pro	C00148	11.446 ± 0.005	0.046	0.010	0.05–50	0.997	105.0

* LOD was defined as the analyte amount with an *S/N* ratio = 3. ** The lowest concentration in the experimentally confirmed linear range was defined as LOQ (*S/N* > 10). *** The Recovery ratio means value obtained the addition–recovery test. The procedure of the test was described in Materials and Methods.

## Data Availability

The data presented in this study are available within the article and Supplementary Materials. All tables and figures are original and have not been taken from any publication.

## Supplementary Materials

The following supporting information can be downloaded at https://www.mdpi.com/article/10.3390/metabo12090807/s1: Figure S1: Derivatization of amino acids for fluorescence detection. Figure S2: Repeatability of amino acid quantification using the iPDAQ method. Figure S3: Procedure and calculation example of the standard addition-recovery test. Table S1: Derivatization conditions. Table S2: Profiles of derivatization reaction reagents (iPDAQ). Table S3: Example of the derivatization condition (conventional method).
