# Supplementary material for: In-Needle Pre-Column Derivatization for Amino Acid Quantification (iPDAQ) Using HPLC
Source: Metabolites. 2022 Aug 28;12(9):807. doi: 10.3390/metabo12090807 (PMC9504251; doi:10.3390/metabo12090807)
Supplement: Supplementary file 1 [file metabolites-12-00807-s001.zip › metabolites-1886708-supplementary.pdf]

Supplementary Figures

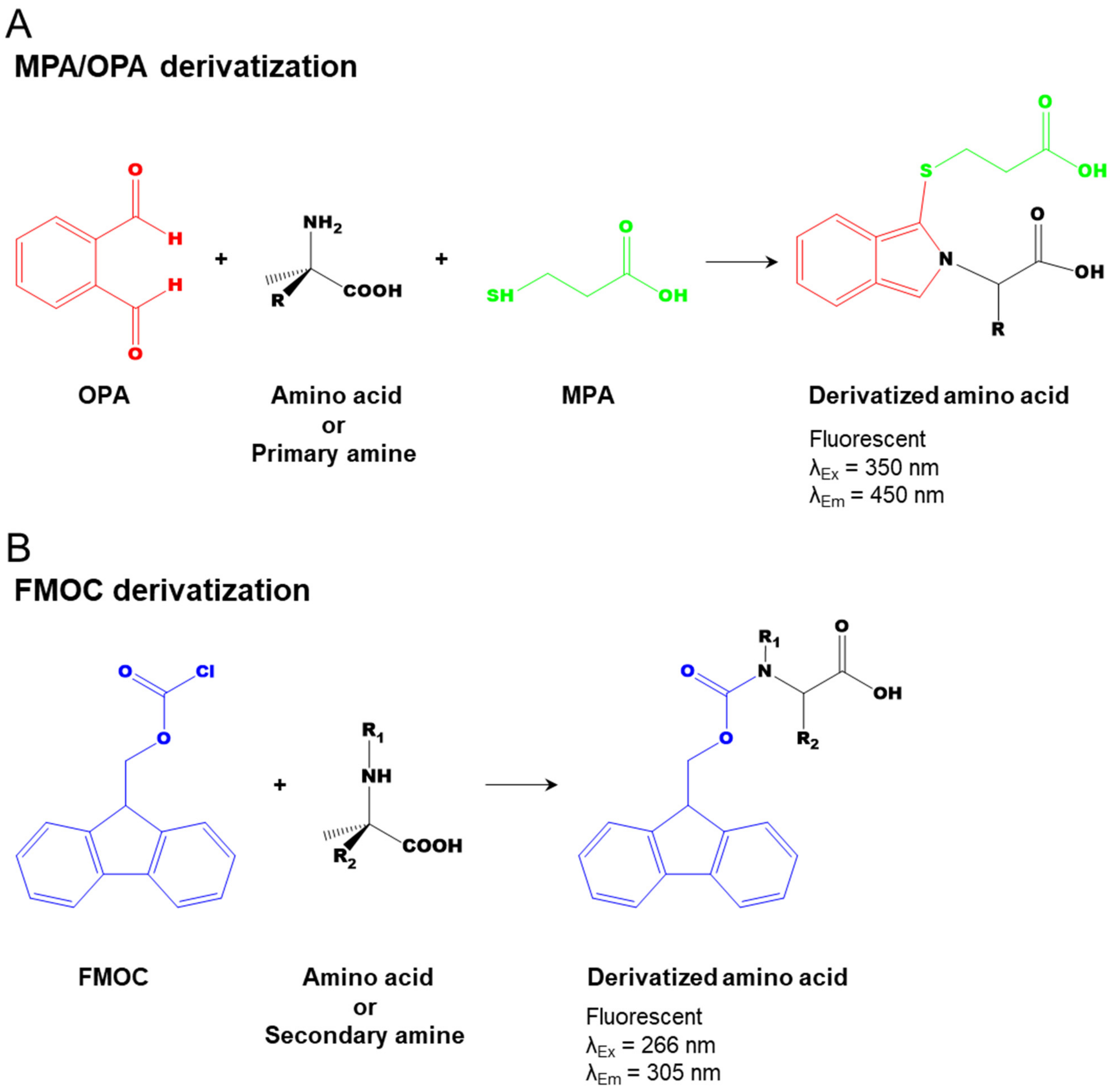

Figure S1. Derivatization of amino acids for fluorescence detection. Reaction in the derivatization of amino acids using (A) MPA/OPA and (B) FMOC.

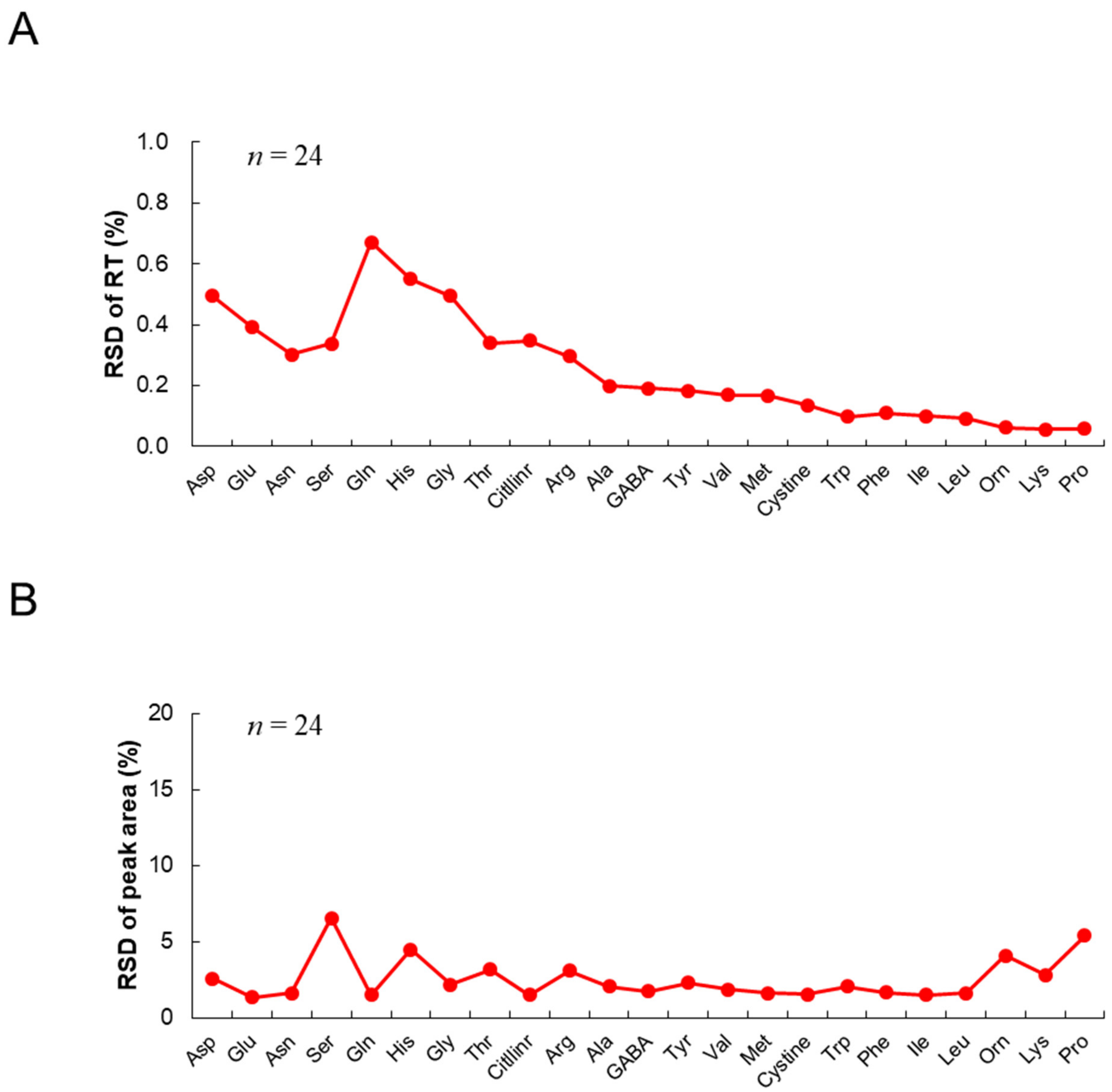

Figure S2. Repeatability of amino acid quantification using the iPDAQ method. (A) Relative standard deviations (RSDs) of retention time (RT). (B) RSDs of peak area. RSD for each amino acid was calculated based on repeat analysis of 10 μM amino acid STD mixture 24 times.

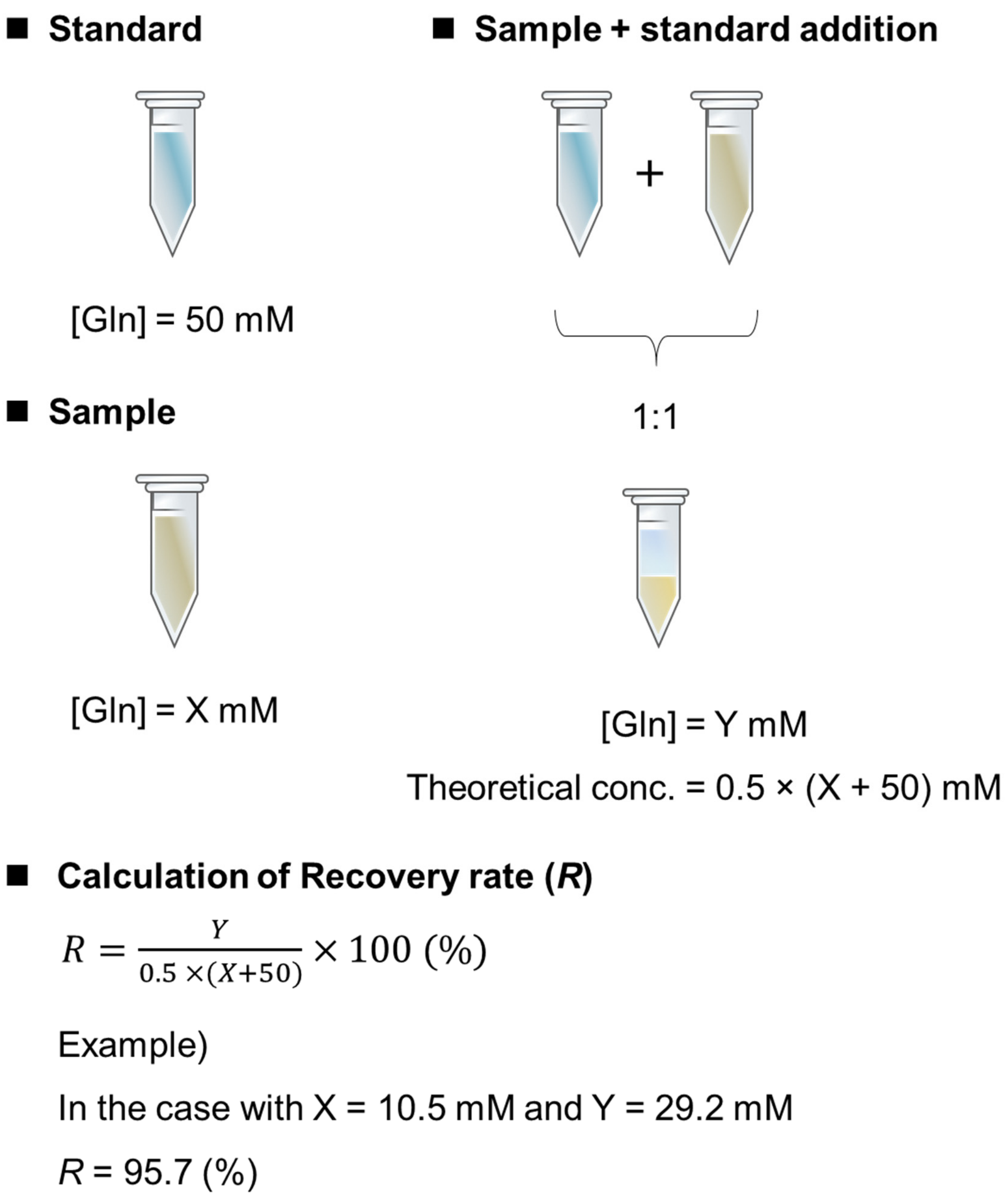

Figure. S3. Procedure and calculation example of the standard addition-recovery test. The recovery rate is calculated as the ratio of the actual value to the theoretical value for the mixture of standards and samples.

Supplementary Tables

Table S1. Derivatization condition

Contents	Parameter
**Derivatization reagents**	
MPA/OPA mixing ratio	1 mL:0.5 mL (2:1)
MPA solution conc. (individual)	0.1% (v/v)
OPA solution conc. (individual)	2.0 mg/mL
FMOC conc.	0.4 mg/mL
**Initialization**	
Air gap	2.5 µL
**Mixing condition**	
Pumping volume	2 µL
Pumping speed	5 µL/s
Mixing time by pumping after MPA/OPA and sample loading	10 times
Mixing time by pumping after FMOC loading	10 times
**Reaction condition for primary amino acids derivatization**	
Total reaction volume	2.0 µL
MPA/OPA mixture volume	1.5 µL
MPA volume (individual)	(1.0 µL)
OPA volume (individual)	(0.5 µL)
Sample volume	0.5 µL
Reaction time for primary amino acid derivatization	0.5 min
**Reaction condition for secondary amino acids derivatization**	
Total reaction volume	2.5 µL
FMOC volume	0.5 µL
Reaction time for secondary amino acid derivatization	2.0 min
**Injection step**	
Phosphate buffer volume	2.5 µL
Injection volume (reaction solution + phosphate buffer)	5.0 µL

Table S2. Profile of derivatization reaction reagents (iPDAQ)

**Reagent**		**MPA**	**OPA**	**FMOC**	**STD**
MW	(g/mol)	106.14	134.13	258.70	-
Conc.	(mM)	11.5	14.9	1.55	0.0250
Loading vol.	(μL)	0.667	0.333	1.00	1.00
Final dilution rate		4.5	9.0	3.0	3.0
Conc. at the 1st reaction	(mM)	3.83	2.48	-	0.0125
Conc. at the 2nd reaction	(mM)	2.55	1.66	0.517	0.00833
Final conc.	(mM)	2.55	1.66	0.515	0.00800
Ratio in the final solution	(vs. analyte)	306	199	62	1
Injection vol.	(μL)		3		
On-column amount	(nmol)	7.65	4.97	1.55	0.0250

Table S3. Example of derivatization condition (conventional method)

Contents	Parameter
**Derivatization reagents**	
MPA solution conc. (individual)	0.1% (v/v)
OPA solution conc. (individual)	2.0 mg/mL
FMOC conc.	0.4 mg/mL
**Initialization**	
Air gap	0.1 μL
**Mixing condition**	
Pumping volume	5 μL
Pumping speed	5 μL/s
Mixing time by pumping after MPA/OPA loading*	10 times
Mixing time by pumping after sample loading*	10 times
Mixing time by pumping after FMOC loading*	10 times
**Reaction time for primary amino acids derivatization**	
Total reaction volume	45.5 μL
MPA/OPA mixture volume	45 μL
MPA volume (individual)	30 μL
OPA volume (individual)	15 μL
Sample volume	0.5 μL
Reaction time for secondary amino acids derivatization	0.5 min
**Reaction time for secondary amino acids derivatization**	
Total reaction volume	50.5 μL
FMOC volume	5 μL
Reaction time for secondary amino acids derivatization	2.0 min
**Injection step**	
Reaction solution loading volume	2.5 μL
Phosphate buffer volume	2.5 μL
Injection volume (reaction solution + phosphate buffer)	5 μL

* The reagents and sample loaded into the needle was mixed by repeating suction and discharge in the needle
